# Effects of Quercetin and Curcumin Combination on Antibacterial, Antioxidant, In Vitro Wound Healing and Migration of Human Dermal Fibroblast Cells

**DOI:** 10.3390/ijms23010142

**Published:** 2021-12-23

**Authors:** Chuda Chittasupho, Amornrat Manthaisong, Siriporn Okonogi, Sarin Tadtong, Weerasak Samee

**Affiliations:** 1 Department of Pharmaceutical Sciences, Faculty of Pharmacy, Chiang Mai University, Mueang, Chiang Mai 50200, Thailand; chuda.c@cmu.ac.th (C.C.); siriporn.okonogi@cmu.ac.th (S.O.); 2 Research Center of Pharmaceutical Nanotechnology, Faculty of Pharmacy, Chiang Mai University, Chiang Mai 50200, Thailand; 3 Department of Pharmaceutical Chemistry, Faculty of Pharmacy, Srinakharinwirot University, Ongkharak, Nahon Nayok 26120, Thailand; amornrat9945@gmail.com; 4 Department of Pharmacognosy, Faculty of Pharmacy, Srinakharinwirot University, Ongkharak, Nahon Nayok 26120, Thailand

**Keywords:** quercetin, curcuminoids, synergistic effect, antibacterial, antioxidant, wound healing

## Abstract

Wound healing impairment due to a postponed, incomplete, or uncoordinated healing process has been a challenging clinical problem. Much research has focused on wound care, particularly on discovery of new therapeutic approaches for acute and chronic wounds. This study aims to evaluate the effect of the combination of quercetin and curcuminoids at three different ratios on the antimicrobial, antioxidant, cell migration and wound healing properties. The antioxidant activities of quercetin, curcuminoids and the mixtures were tested by DPPH and ABTS free radical scavenging assays. The disc diffusion method was performed to determine the antibacterial activities of quercetin, curcuminoids and the mixtures against *S. aureus* and *P. aeruginosa*. The cytotoxicity and cell migratory enhancing effects of quercetin, curcuminoids and the mixtures against human dermal fibroblasts were investigated by MTT assay, scratch assay and Transwell migration assay, respectively. The results showed the synergism of the quercetin and curcuminoid combination to inhibit the growth of *S. aureus* and *P. aeruginosa*, with the inhibition zone ranging from 7.06 ± 0.25 to 8.78 ± 0.38 mm, respectively. The DPPH free radical scavenging assay demonstrated that the combination of quercetin and curcuminoids yielded lower IC_50_ values (15.38–23.70 µg/mL) than curcuminoids alone (25.75 µg/mL). Quercetin and a 3:1 quercetin/curcuminoid mixture at non-toxic concentrations showed the ability to stimulate the migration of fibroblasts across the matrix, whereas only quercetin alone accelerated the wound closure of fibroblasts. In conclusion, the mixture of quercetin and curcuminoids at a 3:1 ratio was the best formulations for use in wound healing due to the antimicrobial, antioxidant and cell-migration-enhancing activities.

## 1. Introduction

The skin provides a protective barrier against physical, thermal, mechanical and chemical injury by preventing the body from dehydration and UV radiation and regulating body temperature [1]. A wound is an injury involving the disruption of external or internal skin integrity. A chronic wound can lead to complications such as bacterial infection [2]. Chronic wounds result in significant problems for patients, healthcare professionals and the government healthcare system. After an injury, wound healing proceeds through four overlapping stages, including coagulation, inflammation, re-epithelization and tissue remodeling [3]. The ultimate goal of wound repair is to achieve tissue integrity and homeostasis. Coagulation involves platelet aggregation, providing a provisional extracellular matrix for cell migration [4]. The inflammation occurs where white blood cells such as phagocytic cells, neutrophils and macrophages migrate to the injured site [5]. At this stage, foreign particles, damaged cells, pathogens and microbes are removed from the wound area. Cytokines are released to promote fibroblast migration and proliferation. In the re-epithelization stage, new blood vessels are formed, so-called angiogenesis or neovascularization, to support perfusion to the newly generated tissues and extracellular matrix [6]. The tissue remodeling phase involves collagen synthesis to strengthen the tissue. At this stage, the wound continues to contract, and fibers are reorganized to form a scar. The goal of wound healing enhancement is to accelerate wound repair, prevent infection, reduce pain, remove dead tissue, provide a moist environment, reduce edema and increase blood flow.

Acute and chronic wounds are susceptible to bacterial infection, leading to systemic infection, which is life-threatening. Bacterial infection also delays the wound healing process, resulting in prolonged inflammation [7]. In chronic wounds, sustained inflammatory responses cause the accumulation of reactive oxygen species (ROS). A low level of ROS plays a critical role in angiogenesis at the wound site and regulates blood perfusion into the wound area. However, excess ROS can act as secondary messengers to recruit lymphoid cells to the wound site, thus preventing the transition of the wound from the inflammatory phase to the proliferative phase [8]. Therefore, a strategy for wound healing acceleration might be designed by applying substances that combine antimicrobial, antioxidant and cell migratory induction. Antibacterial agents can prevent wound infection, and antioxidant substances can maintain ROS levels in the wound area at non-toxic concentrations to improve wound healing. In addition, substances promoting the migration of dermal fibroblasts can accelerate the re-epithelialization phase by increasing epithelial cell migration.

Among the natural polyhydroxy flavonoids, quercetin has strong antioxidant and anti-inflammatory properties to regulate oxidative stress and inflammation, which delay the wound healing process [9]. It has been shown that quercetin can promote the wound healing process by modulating inflammatory cells and increasing fibroblast proliferation while decreasing immune cell infiltration, fibrosis and scar formation, and creating changes in signaling in fibrosis-associated signaling pathways [10,11,12]. A previous report revealed that quercetin accelerated diabetic wound repair by inhibiting inflammatory reactions via modulating macrophage polarization [13]. In addition, quercetin was shown to accelerate cutaneous wound healing in rats by modulating inflammatory and anti-inflammatory cytokines, angiogenic growth factors and antioxidant systems, which might be responsible for the efficient proliferation of cells and increase collagen deposition [12].

The major compounds in turmeric are curcuminoids. Curcuminoids are natural polyphenols consisting of curcumin, demethoxycurcumin and bismethoxycurcumin. Several types of pharmacological activity, such as anti-inflammatory, antimicrobial and antioxidant activities, have been reported for curcuminoids. The principal compound in curcuminoids, curcumin, can enhance the wound healing process by protecting the wound tissue from bacterial infection, reducing inflammation and inducing cell proliferation to help in the regeneration of damaged tissue [14]. An in vivo study showed that curcumin enhanced cutaneous wound healing by increasing fibroblast proliferation, epithelial regeneration, tissue remodeling, granulation, tissue formation and collagen deposition [15].

A combination of antioxidant, antimicrobial and dermal fibroblast migratory stimulating activities may help to promote wound healing and skin regeneration. The purpose of this study was to explore the impact of quercetin, curcuminoids and the combination of quercetin and curcuminoids on inhibiting bacterial growth, antioxidant activity, cell proliferation and cell migration involved in the wound healing process. The results of this study may help to identify the synergistic effect of quercetin and curcuminoids for enhancing the wound healing properties of a single compound.

## 2. Results

### 2.1. Antibacterial Activity of Quercetin, Curcuminoids and Quercetin/Curcuminoid Mixtures

*S. aureus* and *P. aeruginosa* play an important role in cutaneous chronic infected wounds. The antibacterial activities of quercetin, curcuminoids and quercetin/curcuminoid mixtures at 250 µg/mL against *S. aureus* and *P. aeruginosa* were examined using the disc diffusion test. The results of the antibacterial tests are shown in Table 1. The highest inhibition zones against *S. aureus* and *P. aeruginosa* were obtained with the quercetin/curcuminoid mixture at a ratio of 1:1, with inhibition zones of 8.78 ± 0.38 and 8.67 ± 0.00 mm, respectively. Only quercetin/curcuminoid mixtures resulted in the presence of inhibition zones. Quercetin or curcuminoids as single compounds did not demonstrate an inhibition zone for either *S. aureus* or *P. aeruginosa*, indicating the absence of antibacterial activity against these two strains of bacteria. The negative ethanol control showed no inhibitory effect, with inhibition zones of 0 mm, while the positive control (neomycin sulfate 100 µg/mL) showed inhibitory zone diameters ranging from 14.67 ± 0.58 to 16.33 ± 0.58 mm for *S. aureus* inhibition and 12.00 ± 0.00 to 13.33 ± 0.58 mm for *P. aeruginosa* inhibition.

### 2.2. Effects of Quercetin, Curcuminoids and Quercetin/Curcuminoid Mixtures on DPPH and ABTS Free Radical Scavenging

**Figure 1:**
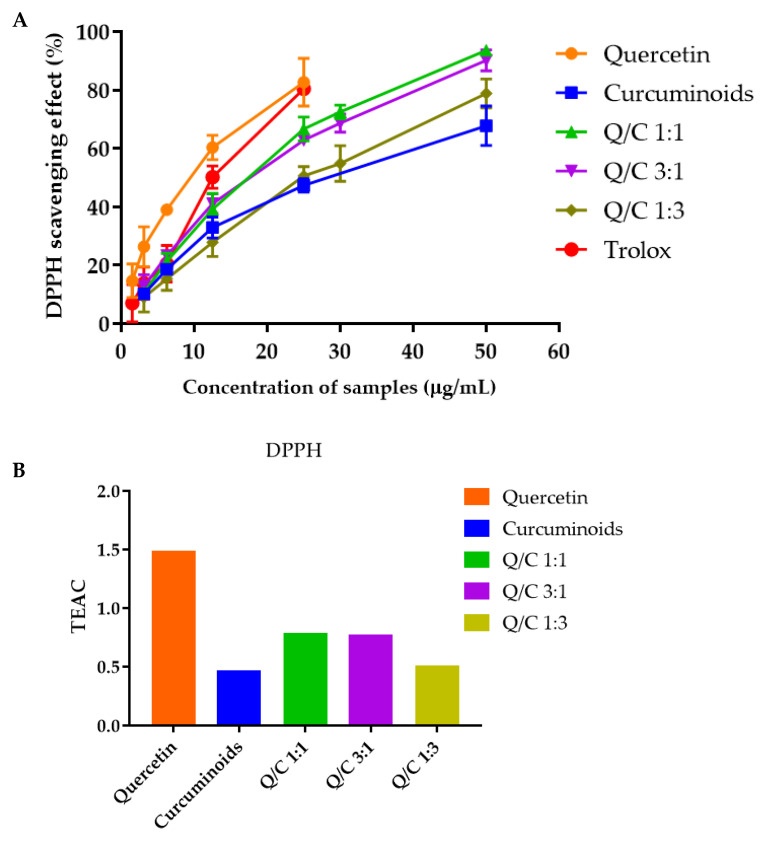
(**A**) DPPH radical scavenging activity of quercetin, curcuminoids and quercetin/curcuminoid mixtures. (**B**) Trolox equivalent antioxidant capacity (TEAC) of quercetin, curcuminoids and quercetin/curcuminoid mixtures.

**Figure 2:**
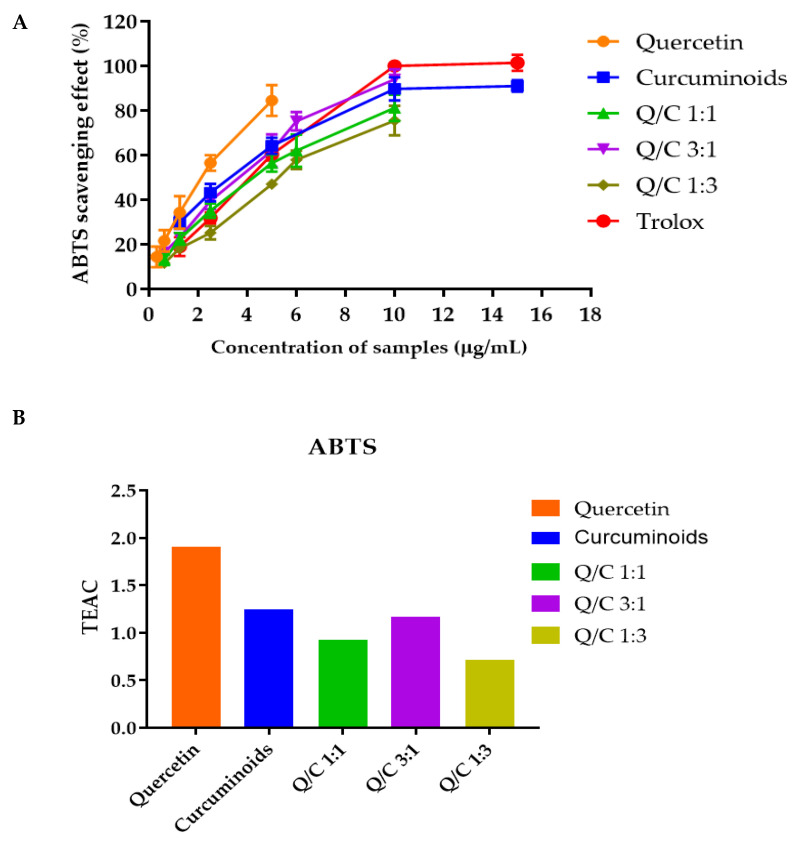
(**A**) ABTS radical scavenging activity of quercetin, curcuminoids and quercetin/curcuminoid mixtures. (**B**) Trolox equivalent antioxidant capacity (TEAC) of quercetin, curcuminoids and quercetin/curcuminoid mixtures.

Quercetin alone exhibited the highest DPPH and ABTS free radical scavenging activities. At a concentration of 25 µg/mL, the DPPH free scavenging activity of quercetin, curcuminoids, Trolox^®^ and 1:1, 3:1 and 1:3 quercetin/curcuminoid mixtures were 82.73, 47.35, 80.43, 66.72, 62.89 and 66.72%, respectively (Figure 1A). The IC_50_ values of quercetin, curcuminoids, Trolox^®^ and the 1:1, 3:1 and 1:3 quercetin/curcuminoid mixtures, obtained from the DPPH radical scavenging assay, were 8.90, 25.26, 12.39, 17.79, 18.96 and 27.14 µg/mL, respectively (Table 2). The TEAC values of quercetin, curcuminoids, Trolox^®^ and the 1:1, 3:1 and 1:3 quercetin/curcuminoid mixtures, determined by the DPPH free radical scavenging assay, were 1.49, 0.47, 0.79, 0.78 and 0.51, respectively (Figure 1B). The ABTS free radical scavenging activities of Trolox, quercetin, curcuminoids and 1:1, 1:3 and 3:1 quercetin/curcuminoid mixtures are shown in Figure 2A. At a concentration of 5 µg/mL, the scavenging activity values of quercetin, curcuminoids, Trolox^®^ and the 1:1, 3:1 and 1:3 quercetin/curcuminoid mixtures were 84.50, 64.29, 60.35, 56.42, 62.07 and 47.07%, respectively (Figure 2A). The IC_50_ values of quercetin, curcuminoids, Trolox^®^ and the 1:1, 3:1 and 1:3 quercetin/curcuminoid mixtures, obtained from the ABTS radical scavenging assay, were 1.83, 2.80, 3.78, 2.98, 4.88 and 3.49 µg/mL, respectively (Table 2). The IC_50_ of quercetin determined by the ABTS assay in this study was 1.83 µg/mL. This result was supported by previous reports. Rusmana et al. reported that the IC_50_ value of quercetin determined by ABTS assay was 1.17 ± 0.02 µg/mL, and the IC_50_ value of quercetin reported by Lee et al. was 1.89 ± 0.33 µg/mL. The TEAC values of quercetin, curcuminoids, Trolox^®^ and 1:1, 3:1 and 1:3 quercetin/curcuminoid mixtures, determined by the ABTS free radical scavenging assay, were 1.91, 1.25, 0.92, 1.17 and 0.71, respectively (Figure 1B).

### 2.3. Cytotoxicity of Quercetin, Curcuminoids and Quercetin/Curcuminoid Mixtures

**Figure 3:**
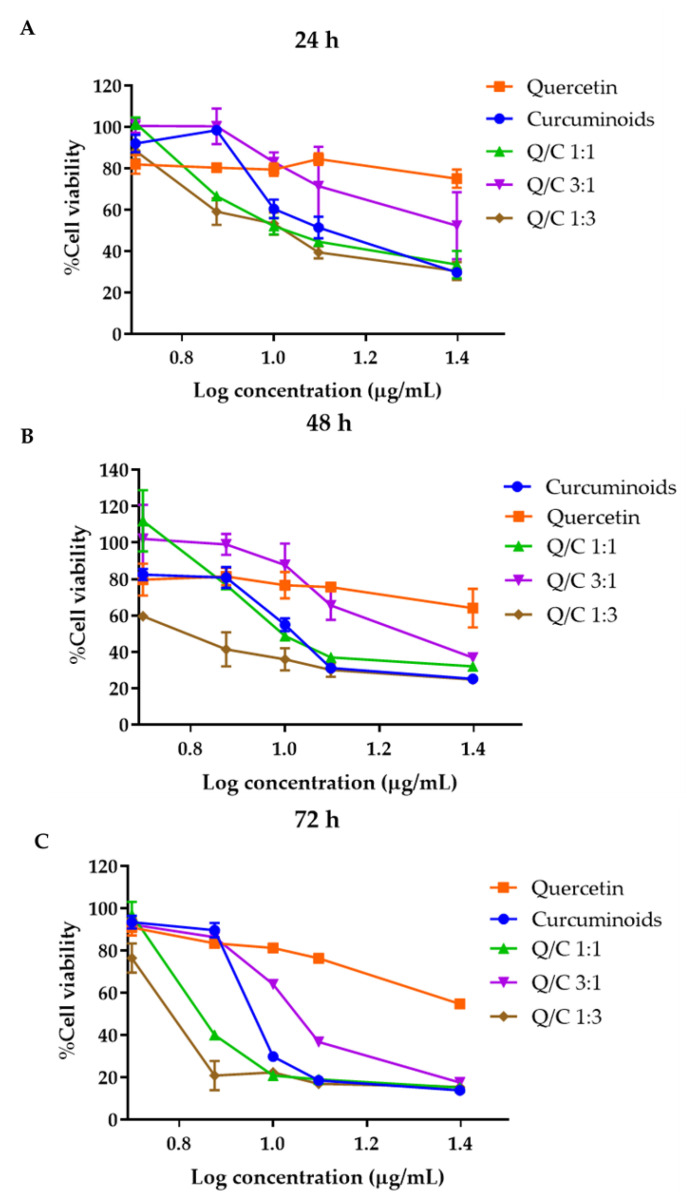
HDFB cell viability after treatment with quercetin, curcuminoids and quercetin/curcuminoid mixtures and further incubated for (**A**) 24 h, (**B**) 48 h and (**C**) 72 h.

The cytotoxicity of quercetin, curcuminoids and quercetin/curcuminoid mixtures at concentrations ranging from 5 to 25 µg/mL was investigated on a human dermal fibroblast cell line by using the MTT assay. The effects of quercetin, curcuminoids and quercetin/curcuminoid mixtures on the human dermal fibroblast cell line were evaluated after 24, 48 and 72 h of cell exposure to the compounds. Cell viability decreased with the increase in the concentration of all compounds. At 7.5 µg/mL, quercetin, curcuminoids and the quercetin/curcuminoid mixture at a 3:1 ratio yielded more than 80% cell viability in 24, 48 and 72 h (Figure 3). Interestingly, cells treated with the 1:1 and 1:3 quercetin/curcuminoid mixtures had less than 80% cell viability after 24, 48 and 72 h of exposure. The cell viability decreased in a time- and dose-dependent manner. The average %viability of cells treated with 1.25% *v/v* DMSO (solvent control) after 24, 48 and 72 h was 93.93 ± 19.29%, 85.93 ± 15.59% and 101.69 ± 15.99%, respectively. The statistical analysis results showed that there was no significant difference in the %cell viability at 24, 48 and 72 h. According to ISO 10993-5, percentages of cell viability above 80% are considered to indicate non-cytotoxicity [16,17]. The IC_50_ values of quercetin, curcuminoids and quercetin/curcuminoid mixtures were calculated after 24, 48 and 72 h of treatment (Table 3). The IC_50_ values of each compound and the mixture decreased with incubation time, suggesting that quercetin, curcuminoids and the mixtures reduced human dermal fibroblast cell viability in a dose- and time-dependent manner.

### 2.4. Effects of Quercetin, Curcuminoids and Quercetin/Curcuminoid Mixtures on Wound Closure

**Figure 4:**
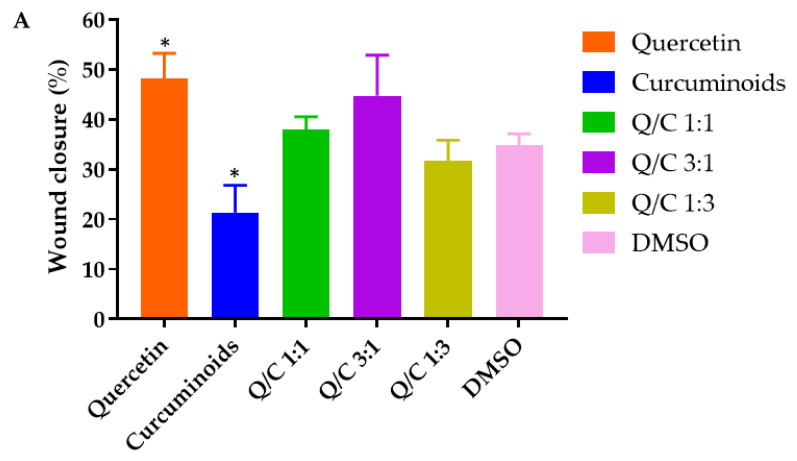

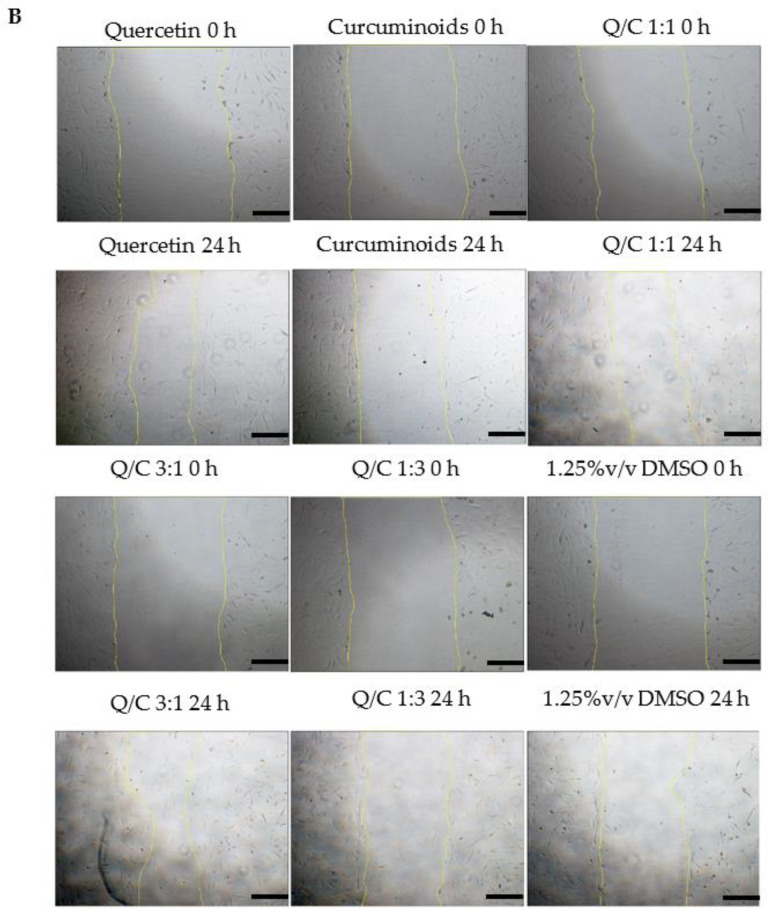
(**A**) Quantitative analysis of the migration area reported as %wound closure. (**B**) Representative images of HDFB cells in a wound scratch assay. The images were taken immediately after the scratches had been made and then after 24 h in the presence and absence of quercetin, curcuminoids and quercetin/curcuminoid mixtures. Data represent mean ± S.D. (*n* = 3). * *p* < 0.05 for comparison with control (DMSO). Scale bars represent 200 µm.

Cell migration is critical for the wound healing process. The wound healing potential of quercetin, curcuminoids and the quercetin/curcuminoid mixtures was investigated by evaluating the ability of the compounds to induce HDFB cell migration in comparison to the solvent control (1.25% DMSO in serum-free DMEM) using a scratch assay. The results showed that %wound closure was determined by the measurement of cell-free areas of the cell monolayer after exposure to the treatments for 24 h. The %wound closure values of HDFB cells treated with quercetin, curcuminoids, 1:1 quercetin/curcuminoids, 3:1 quercetin/curcuminoids, 1:3 quercetin/curcuminoids and the serum-free DMEM control were 48.1, 21.37, 37.89, 44.7, 31.71 and 34.84%, respectively (Figure 4).

### 2.5. Effects of Quercetin, Curcuminoids and Quercetin/Curcuminoid Mixtures on HDFB Cell Migration

**Figure 5:**
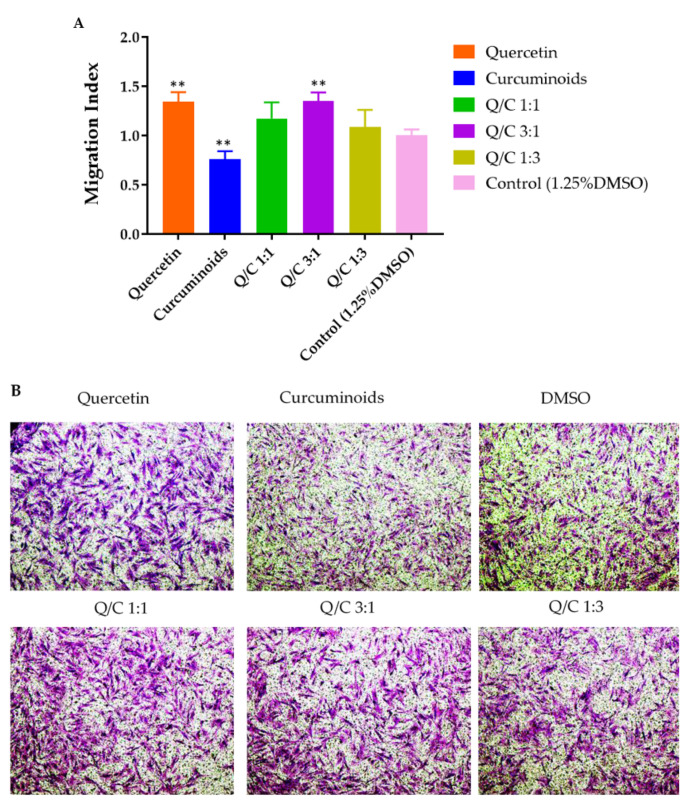
Transwell migration of HDFB cells. (**A**) Migration index of cells treated with quercetin, curcuminoids and quercetin/curcuminoid mixture. (**B**) Representative images showing migrating cells per field after treatment with quercetin, curcuminoids and quercetin/curcuminoid mixture. Data represent mean ± S.D. (*n* = 3), and ** *p* < 0.01 in comparison with control (1.25% DMSO in serum-free DMEM). Scale bars represent 100 µm.

To determine the effects of quercetin, curcuminoids and quercetin/curcuminoid mixtures on HDFB cell migration, cells were treated with compounds at a non-toxic concentration (7.5 µg/mL) in serum-free media. The migration index was calculated as the fold of the average number of migrated cells increased over the control (serum-free medium). The migration index values of HDFB cells treated with quercetin, curcuminoids, 1:1 quercetin/curcuminoids, 3:1 quercetin/curcuminoids, 1:3 quercetin/curcuminoids and the serum-free DMEM control were 1.34, 0.76, 1.17, 1.35, 1.09 and 1.00, respectively (Figure 5).

## 3. Discussion

Quercetin and curcuminoids are bioactive compounds that display several types of pharmacological activity, including antioxidant, antimicrobial, anti-inflammatory and wound healing enhancing, etc. [18].

The antibacterial activities of plant flavonoids against Gram-positive bacteria—in particular, *S. aureus*—have been widely investigated. In contrast, reports of the inhibitory activity of flavonoids against Gram-negative bacteria are limited. In our study, quercetin and curcuminoids alone did not show antimicrobial activities, whereas a combination of quercetin and curcuminoids enhanced the antibacterial activities of *S. aureus* (Gram-positive bacteria) and *P. aeruginosa* (Gram-negative bacteria), suggesting the use of quercetin/curcuminoid mixtures for preventing bacterial infection in wounds. The antibacterial activity of quercetin is limited to certain bacterial species. Hirai et al. reported the antibacterial activities of quercetin against *S. aureus* and *S. epidermidis* at relatively high concentrations compared with common antibiotics. It has been reported that *P. aeruginosa,* *Streptococcus* spp., *E. faecalis*, *E. coli* and other Gram-positive cocci are not susceptible to quercetin up to 50 µM. Curcuminoids have shown antibacterial activities against *S. aureus*, *S. epidermidis* and *S. intermidis* but have not shown inhibitory effects on Gram-negative bacteria, including *Vibrio* spp., *Salmonella* spp. and *E. coli*. In another study, curcuminoids were shown to inhibit the growth of *S. aureus* but not *E. coli* [19]. Although most Gram-negative bacteria show low sensitivity to curcuminoids, there have been reports of the inhibitory effect of curcumin against *P. aeruginosa* [20]. Our results showed that quercetin and curcuminoids alone did not inhibit the growth of *S. aureus* and *P. aeruginosa*. This might be due to several factors affecting the results of the disc diffusion assay. For example, the inhibition zones can be affected by the rate of diffusion of the compound [21]. In addition, it has been reported that the MIC values of each flavonoid against each bacterial species depend on its lipophilicity. Quercetin and curcuminoids are slightly soluble in water, which may limit the diffusion rate of the compounds. The mechanisms of the antibacterial activity of flavonoids are the damage of the phospholipid bilayer of the bacterial cell wall and cell membrane, leading to an increase in the permeability of this structure and the inhibition of ATP synthesis [22,23]. Combining quercetin and curcuminoids enhanced their antimicrobial activities against *S. aureus* and *P. aeruginosa*, indicating a synergism between the two polyphenolic compounds. The synergistic antimicrobial effects of the quercetin and curcumin combination were reported. Combining these two phenolic compounds led to enhanced antibacterial activity against Methicillin-resistant Staphylococcus aureus (MRSA) [24]. The MIC values of the quercetin and curcumin combination were lower than those of the single compounds. The synergistic effect of quercetin and curcumin was probably due to the fact that both compounds have different modes of action to kill bacteria. The mechanism of the antimicrobial activity of quercetin is the inhibition of cell wall synthesis, inhibition of DNA gyrase and impairment of cell motility [25]. Curcumin kills bacteria by binding with the FtsZ protein, which plays a significant role in producing a new cell wall during cell division [26].

Polyphenols and flavonoids exert antioxidant mechanisms through several pathways, including direct antioxidant action, metal chelation or the regeneration of endogenous antioxidants. The combination of antioxidants can protect against vulnerability to other antioxidants and synergistically potentiate their antioxidant properties [27]. Greater metal chelation resulting in a greater antioxidant effect by combining curcumin and quercetin has been previously reported [28]. In this study, the potential for the synergy of quercetin and curcuminoids in terms of free radical scavenging was investigated. The antioxidant capacity of quercetin, curcuminoids and the mixtures was evaluated by determining the free radical scavenging capacity with respect to Trolox^®^ (TEAC values). The results showed that quercetin exhibited a TEAC value greater than 1 for both the DPPH and ABTS assays, while quercetin, curcuminoids and the 3:1 quercetin/curcuminoid mixture had TEAC values greater than 1 for the ABTS assay, suggesting that these compounds have higher antioxidant capacity compared with Trolox^®^. The results of antioxidant activity demonstrated that quercetin had higher free radical scavenging activity compared with curcuminoids. Quercetin is a natural flavonoid that reacts with a free radical by donating a proton, and the resulting unpaired electron is delocalized by resonance, making the quercetin radical unreactive. Quercetin is a strong antioxidant due to its ability to scavenge free radicals and bind transition metal ions [29]. Curcuminoids are a natural polyphenol compound including curcumin, demethoxycurcumin and bisdemethoxycurcumin. Each component of a curcuminoid exhibits a different level of antioxidant activity [30]. The antioxidant activity of curcumin results from ortho-methoxyphenol groups, which form intramolecular hydrogen bonding with phenolic hydrogen, facilitating proton abstraction from ortho-methoxyphenols [31]. A decrease in or absence of methoxy groups of curcumin causes a significant decrease in antioxidant activity. Sompran et al. demonstrated that demethoxy derivatives of curcumin showed less antioxidant activity compared with curcumin, while bisdemethoxycurcumin had negligible antioxidant activity. The decreasing antioxidant activities of demethoxycurcumin and bisdemethoxycurcumin could be caused by the lack of an ortho-methoxy group [32]. Therefore, the ratio of curcumin, dimethoxycurcumin and bisdemethoxycurcumin was significant to the antioxidant activity of curcuminoids. 

Compared to a single compound, a mixture of quercetin and curcuminoids did not have additive or synergistic free radical scavenging activity. In contrast, increasing the ratio of curcuminoids in the quercetin/curcuminoid mixture resulted in a decrease in the ABTS free radical scavenging activity. The possible reasons for these results might be the lower antioxidant activity of curcuminoids compared with quercetin. Another possibility was that quercetin might react with curcuminoids instead of reacting with DPPH or ABTS, forming a quercetin–curcuminoid adduct, which was not reactive for free radical scavenging [33]. Boots et al. have shown the adduct formation between quercetin and glutathione. Although the adduct was reversible, it was stable within the time of the free radical scavenging measurement and caused the subadditivity of both antioxidants [34]. The antagonistic effect of combined polyphenols on antioxidant activity was previously demonstrated. Murakami et al. reported that the radical scavenging activity of quercetin in combination with catechin or epicatechin was balanced due to their phenolic interaction during oxidation [35]. These results are in agreement with those of Lacopini et al., reporting that the interactions of phenolic compounds in a mixture affect the total antioxidant capacity of the mixture [36]. 

Human dermal fibroblast cells are widely known to play an important role in wound healing, particularly at the re-epithelization stage. The cell viability assay showed that quercetin possessed the highest IC_50_ value against fibroblasts. In contrast, curcuminoids exhibited higher cytotoxicity to human dermal fibroblasts in a dose- and time-dependent manner. The IC_50_ values of the 1:3 quercetin/curcuminoid and 1:1 quercetin/curcuminoid mixtures were lower than that of curcuminoids alone at all incubation time points. The increasing ratio of curcuminoids increased the cytotoxicity of the mixture. These results indicated the synergistic cytotoxic effects of the mixture of quercetin and curcuminoids against human dermal fibroblast cells. The effects of quercetin on human skin fibroblast cell viability and proliferation are in agreement with previous reports. Paliwal et al. demonstrated that quercetin up to 50 µM did not show cytotoxicity against human normal skin fibroblasts and skin cancer cells after 48 h of incubation [37]. Quercetin at 6.25, 12.5 and 25 µM showed no effect on proliferation in human fibroblast cells [38]. Kim et al. demonstrated that quercetin strongly induced mitochondrial reactive oxygen species (ROS) in human embryonic stem cells, but not in human dermal fibroblasts [39]. In contrast, curcumin, demethoxycurcumin and bisdemethoxycurcumin showed cytotoxicity to keratinocytes and fibroblasts at a concentration higher than 50 µM after treatment for 48 h [40]. Curcumin at a concentration higher than 20 µM has been shown to induce decreased human dermal fibroblast cell viability to 57% in a control and a strong inhibition of cell proliferation after 24 h of exposure to 10 and 20 µM of curcumin [41]. In this study, we found that increasing quercetin in the mixture to 3:1 increased the IC_50_ value of curcuminoids, suggesting that a lower amount of curcuminoids in the mixture decreased the cytotoxicity of the mixture. Scharstuhl et al. reported that curcuminoids (25 µM) were shown to cause fibroblast apoptosis by inducing ROS generation. They also found that the co-administration of other antioxidants, including N-acetyl-l-cysteine (NAC), biliverdin or bilirudin, completely blocked ROS generation and inhibited cell apoptosis [42]. Bao et al. reported that quercetin exhibited a protective effect against oxidative stress-induced apoptosis in rat pheochromocytoma cells [43]. More studies are required to identify whether the combination of quercetin and curcuminoids at 1:1 and 1:3 ratios contributes toward the antioxidant–ROS scavenging activity in HDFB cells. 

HDFB cells treated with quercetin (7.5 µg/mL) had a significantly higher wound closure rate than cells treated with the control. Quercetin has been shown to accelerate wound closure in diabetic rats by increasing the expression of IL-10, VEGF and TGF-b1 in granulation tissue formation in the diabetic wound healing process [13]. In addition, several studies indicated that quercetin enhanced wound healing by increasing surface αV integrin and decreasing β1 integrin, resulting in fibroblast cell migration, proliferation and extracellular matrix production, without impairing or improving cell proliferation or survival [10,44]. In our study, curcuminoids significantly reduced the cell migration rate compared with the control. The slower rate of HDFB cell migration after exposure with curcuminoids may be due to the toxicity of curcuminoids against human dermal fibroblast cells or due to a reduction in protein synthesis [45]. Although several reports have suggested the in vivo wound healing properties of curcumin, Topman et al. reported that curcumin did not induce a significant effect on the migration kinematics of cultured fibroblasts, suggesting that curcumin is not involved in the migration of fibroblasts to the wound area in vitro. The fibroblast migration in vivo was influenced by various factors. For example, the infiltration of fibroblasts to the wound area in vivo was promoted by the cytokines released by the inflammatory cells. The migrating cells then differentiated into microfibroblasts during the formation of granulation tissue [46]. The combination of the two compounds at any ratio did not increase or decrease the wound closure rate compared with the control. These results suggest that the wound closure enhancing effect of quercetin might be canceled out in the presence of curcuminoids. The results of Transwell cell migration demonstrated that quercetin and the 3:1 quercetin/curcuminoid mixture significantly induced HDFB migration toward crossing the matrix, compared with the medium control. Curcuminoids were found to decrease the migration of HDFB cells significantly. These results indicate that quercetin induced HDFB cells to close the wound or cross the matrix, whereas curcuminoids inhibited wound closure and the matrix crossing of the cells. The combination of quercetin and curcuminoids did not show a synergistic effect on cell migration toward enhancing wound closure and matrix crossing. In contrast, an increasing ratio of curcuminoids in the mixture (1:1 and 1:3 ratios) resulted in a decrease in HDFB cell migration, which might be related to the cytotoxicity of the quercetin and curcuminoid mixture. Thus, our results suggest that increasing the ratio of quercetin in the quercetin/curcuminoid mixtures to 3:1 can protect HDFB cells from the cytotoxicity of curcuminoids and enhance wound closure, cell matrix crossing and antibacterial activity.

## 4. Materials and Methods

Firstly, 2,2′-azino-bis (3-ethylbenzthiazoline-6-sulphonic acid (ABTS)), 2,2-diphenyl-1- picrylhydrazyl (DPPH), quercetin, curcuminoids and Trolox^®^ were purchased from Sigma Aldrich (St. Louise, MO, USA). Absolute ethanol was purchased from Amresco Inc. (Solon, OH, USA). Fetal bovine serum (FBS), 3-(4,5-dimethylthiazol-2yl)-2,5-diphenyl tetrazolium bromide (MTT), sodium pyruvate, 0.25% trypsin–EDTA and tryptic soy broth (TSB) were purchased from Gibco (Thermo Fisher Scientific, Inc., Waltham, MA, USA). Dulbecco’s modified Eagle medium (DMEM) was purchased from Corning (Glendale, AZ, USA). Dimethyl sulfoxide and (DMSO) and potassium persulfate (K_2_S_2_O_8_) were purchased from Unilab (Sydney, Australia). Agar was obtained from Merck (Darmstadt, Germany). Neomycin sulfate was purchased from Sichuan Long March Pharmaceutical Co., Ltd. (Sichuan, China). Beeswax, lecithin and Carbopol 940^®^ were purchased from Namsiang International Co., Ltd. (Bangkok, Thailand). *Staphylococcus aureus* DMST8013 (ATCC6538) and *Pseudomonas aeruginosa* DMST15501 (ATCC9027) were purchased from the Department of Medical Sciences in the Ministry of Public Health (Bangkok, Thailand).

### 4.1. Determination of Antibacterial Activities of Quercetin, Curcuminoids and Quercetin/Curcuminoid Mixture

The antibacterial activities of quercetin, curcuminoids and the quercetin/curcuminoid mixture at ratios of 1:1, 3:1 and 1:3 at a 250 µg/mL concentration were investigated by the disc diffusion method. Agar plates were prepared by dissolving tryptic soy broth (3 g) and agar (1.5 g) in deionized water (100 mL). The test organisms, *S. aureus* and *P. aeruginosa*, were swabbed over the surface of the solidified agar plates using a sterile swab spreader. Then, the discs containing 20 µL positive control (neomycin sulfate 100 µg/mL), quercetin, curcuminoids, 1:1, 3:1 and 1:3 quercetin/curcuminoids mixture and negative control (ethanol) were impregnated on the inoculated agar plates and incubated at 37 °C for 18 h, followed by the measurement of the inhibition zone formed by the test bacteria around the discs. The inhibition zones were measured using a vernier caliper.

### 4.2. In Vitro Antioxidant Assay by DPPH Method

Quercetin solution in ethanol (1.5–25 μg/mL), curcuminoid solution in ethanol (3.125–50 μg/mL), 1:1, 3:1 and 1:3 quercetin/curcuminoid mixture in ethanol (3.125–50 μg/mL) and Trolox^®^ solution in ethanol (1.5–25 μg/mL) were added to a 96-well plate (100 µL/well). The DPPH ethanolic solution (0.6 mM, 100 µL) was added to the sample and control. Mixtures were shaken and incubated at room temperature for 30 min in the dark. The absorbance of samples, Trolox^®^, blank control of ethanol and blank control of sample was measured at 520 nm using a UV–Vis spectrophotometer microplate reader (Spectramax, San Jose, CA, USA). The inhibition ratio (%) was obtained from Equation (1).

(1) $$\mathrm{DPPH}\;\mathrm{scavenging}\;\mathrm{effect}\;(\%)=\frac{\mathrm{Ablank}\;-\;(\mathrm{ASample}\;-\;\mathrm{Ablank}\;\mathrm{sample})}{\mathrm{Ablank}\;}\times 100\%$$

The Trolox equivalent antioxidant capacity (TEAC) was calculated to compare the antioxidant capacity of quercetin, curcuminoids and 1:1, 3:1 and 1:3 quercetin/curcuminoid mixtures with the standard Trolox^®^ by Equation (2).

(2) $$\mathrm{TEAC}=\frac{\mathrm{IC}50\;\mathrm{trolox}}{\mathrm{IC}50\;\mathrm{sample}}$$

where IC_50trolox_ is the half-maximal inhibitory concentration of Trolox and IC_50sample_ is the half-maximal inhibitory concentration of the sample.

### 4.3. In Vitro Antioxidant Assay by ABTS Method

Quercetin solution in ethanol (0.3125–5 µg/mL), curcuminoid solution in ethanol (1.25–15 µg/mL), 1:1, 3:1 and 1:3 quercetin/curcuminoid mixtures in ethanol (0.625–10 µg/mL) and Trolox^®^ solution in ethanol (1.25–15 µg/mL) were added to a 96-well plate (20 µL/well). The ABTS radical solution was prepared by mixing 7 mM ABTS solution and 2.45 mM K_2_S_2_O_8_ solution at a 1:1 ratio in the dark for 24 h, followed by dilution with ethanol at a 1:19 ratio. The ABTS radical solution (180 µL) was added to the sample solution or control and incubated at 15 min at room temperature. The absorbance of samples, Trolox^®^, blank control of ethanol and blank control of sample was measured at 735 nm using a UV–Vis spectrophotometer microplate reader (Spectramax, San Jose, CA, USA). The inhibition ratio (%) was obtained from Equation (3).

(3) $$\%\;\mathrm{Inhibition}=\frac{\mathrm{Ablank}\;-\;(\mathrm{ASample}\;-\;\mathrm{Ablank}\;\mathrm{sample})}{\mathrm{Ablank}\;}\times 100\%$$

The Trolox equivalent antioxidant capacity (TEAC) was calculated to compare the antioxidant capacity of quercetin, curcuminoids and 1:1, 3:1 and 1:3 quercetin/curcuminoid mixtures with the standard Trolox by Equation (4).

(4) $$\mathrm{TEAC}=\frac{\mathrm{IC}50\;\mathrm{trolox}\;(µg/\mathrm{mL})}{\mathrm{IC}50\;\mathrm{sample}\;(µg/\mathrm{mL})}$$

where IC_50trolox_ is the half-maximal inhibitory concentration of Trolox and IC_50sample_ is the half-maximal inhibitory concentration of the sample.

### 4.4. Cell Culture

Human dermal fibroblasts (HDFB) were cultured in DMEM supplemented with 10% FBS, 2 mM L-glutamine, 100 IU/mL penicillin and 0.1 mg/mL streptomycin at 5% CO_2_ and 37 °C. At 80% confluence, the cells were trypsinized using 0.25% trypsin and seeded in 96-well plates and 24-well plates for cell viability and scratch assay, respectively.

### 4.5. Cell Viability Assay

The cytotoxicity of quercetin, curcuminoids and 1:1, 3:1 and 1:3 quercetin/curcuminoid mixtures was determined by the MTT assay. HDFB cells (80,000 cells/mL) were seeded in 96-well plates (100 µL/well) and cultured for 24 h. The medium from each well was removed. Quercetin, curcuminoids and 1:1, 3:1 and 1:3 quercetin/curcuminoid mixtures dissolved in 1.25%DMSO and serum-free DMEM at concentrations of 5, 7.5, 10, 12.5 and 25 µg/mL were added to the cells and incubated at 37 °C for 24 h. Cells were treated with 1.25% DMSO as a solvent control. After incubation, cells were washed three times with PBS, pH 7.4. Cells were further incubated with MTT (0.5 mg/mL) in culture medium for 2 h at 37 °C, 5% CO_2_. Then, the reagent was removed, and DMSO (100 µL/well) was added to solubilize the formazan crystals. The absorbance was measured at 550 nm. The absorbance values were related to the number of viable cells. The cell viability percentage was calculated using Equation (5), where the control was the viability of untreated cells. The IC_50_ was calculated based on non-linear regression analysis The IC_50_ value of %cell viability refers to the concentration of samples required to bring the curve down to a point halfway between the maximal and the minimal plateaus of the curve, which was calculated from the dose–response curve of %cell viability and log concentration by GraphPad Prism 7.

(5) $$\mathrm{Cell}\;\mathrm{viability}\;(\%)=\frac{A550\;\mathrm{of}\;\mathrm{tested}\;\mathrm{cells}}{A550\;\mathrm{of}\;\mathrm{control}}\times 100\%\;$$

### 4.6. In Vitro Scratch Assay

The stimulatory effect of quercetin, curcuminoids and 1:1, 3:1 and 1:3 quercetin/curcuminoid mixtures on the migration of HDFB cells was determined by scratch assay [47]. HDFB cells were plated into a 24-well plate at a concentration of 3 × 10^5^ cells/mL (500 µL/well) and incubated for 24 h at 37 °C, 5% CO_2_. After the cells were attached to the plates, the culture medium was removed, and the adherent cell layer was scratched with a SPLScar™ Scratcher. Cells were then treated with serum-free DMEM containing 7.5 µg/mL of quercetin, curcuminoids and 1:1, 3:1 and 1:3 quercetin/curcuminoid mixture in 1.25% *v/v* DMSO (500 µL/well). Control cells received only serum-free DMEM containing 1.25% *v/v* DMSO. Images of the scratch area were captured at 0 h (before sample treatment) and 24 h after incubation with the test materials using a built-in camera in the microscope (40× magnification). The wound closure (%) was calculated by Equation (6) as follows.

(6) $$\mathrm{Wound}\;\mathrm{closure}\;\left(\%\right)\frac{\mathrm{Area}\;\mathrm{between}\;\mathrm{cells}\;\mathrm{at}\;0\;h-\mathrm{Area}\;\mathrm{between}\;\mathrm{cells}\;\mathrm{at}\;24\;h\;}{\mathrm{Area}\;\mathrm{between}\;\mathrm{cells}\;\mathrm{at}\;0\;h}\times 100\%\;$$

### 4.7. Transwell Migration Assay

The Transwell migration assay was performed using a 6.5 mm diameter chamber with an 8.0 μm pore polycarbonate filter (Transwell, 24 well cell culture) [48,49]. HDFB cells at a density of 1 × 10^5^ cells/mL were incubated with quercetin, curcuminoids and 1:1, 3:1 and 1:3 quercetin/curcuminoid mixtures at 7.5 µg/mL or serum free DMEM for 1 h at 37 °C, 5% CO_2_. After incubation with samples, HDFB cells were washed, resuspended in serum-free medium and 0.2 mL cell suspension was added to the upper chamber. Then, 0.7 mL 10% FBS DMEM was added to the lower chamber. The chambers were incubated for 24 h at 37 °C, 5% CO_2_. After incubation, the upper surfaces of the filters were scraped twice with cotton swabs to remove non-migrating cells. The filters were fixed with 4% paraformaldehyde and stained with 0.1% crystal violet. The number of migrating cells in five high-power fields per filter was counted microscopically at ×40 magnification. Data were normalized as the migration index, calculated by the number of migrating cells in the Transwell chamber/the number of migrating cells in the lower chamber.

### 4.8. Statistical Analysis

Statistical analysis of data was completed using analysis of variance (one-way ANOVA), followed by Tukey as a post-hoc test to assess the significance of differences. To check the significance of the difference between the means of the two groups, a *t*-test was performed. In all cases, a value of *p* < 0.05 was considered statistically significant. All data represent mean ± S.D., *n* = 3 replicates.

## 5. Conclusions

The mixtures of quercetin and curcuminoids at 1:1, 3:1 and 1:3 ratios showed enhanced antimicrobial activities against *S. aureus* and *P. aeruginosa*. Quercetin exhibited stronger antioxidant activity compared to curcuminoids. The combination of quercetin and curcuminoids had antioxidant activity between that of quercetin and curcuminoids. At a non-toxic concentration, quercetin alone could accelerate human dermal fibroblast cell migration to close the wound. Quercetin and the quercetin/curcuminoid mixture at a 3:1 ratio increased the migration rate of cells to cross the matrix. These results provide scientific evidence for the application of a quercetin/curcuminoid combination for wound healing. In conclusion, quercetin alone and the mixture of quercetin and curcuminoids at a 3:1 ratio are the best formulations for use in wound healing due to the antimicrobial, antioxidant and cell migration enhancing activities.

## Figures and Tables

**Table 1:** Zone of inhibition (mm) of quercetin, curcuminoids and quercetin/curcuminoid mixtures at 1:1, 3:1 and 1:3 ratios against *S. aureus* (DMST 8013) and *P. aeruginosa* (DMST 15501).

Sample	Concentration (µg/mL)	*S. aureus* (DMST 8013) (mm)	*P. aeruginosa* (DMST 15501) (mm)
Quercetin	250	0	0
Curcuminoids	250	0	0
Q:C (1:1)	250	8.78 ± 0.38	8.67 ± 0.00
Q:C (3:1)	250	8.33 ± 0.33	7.67 ± 0.33
Q:C (1:3)	250	7.22 ± 0.19	7.06 ± 0.25
Neomycin sulfate (Positive control)	100	15.33 ± 0.82	12.60 ± 0.63

**Table 2:** The IC_50_ values of quercetin, curcuminoids and 1:1, 3:1 and 1:3 quercetin/curcuminoid mixtures obtained from DPPH and ABTS assay.

	DPPH (µg/mL)	ABTS (µg/mL)
Quercetin	8.14	1.83
Curcuminoids	25.75	2.80
Quercetin/Curcuminoids 1:1	15.38	3.78
Quercetin/Curcuminoids 3:1	15.59	2.98
Quercetin/Curcuminoids 1:3	23.70	4.88
Trolox	12.10	3.49

**Table 3:** The IC_50_ values of quercetin, curcuminoids and 1:1, 3:1 and 1:3 quercetin/curcuminoid mixtures obtained from MTT assay.

	24 h	48 h	72 h
Quercetin	>25	>25	29.44
Curcuminoids	14.57	11.11	9.3
Quercetin/Curcuminoids 1:1	12.6	11.52	7.437
Quercetin/Curcuminoids 3:1	24.78	19.22	11.69
Quercetin/Curcuminoids 1:3	11.24	6.694	6.271

## Data Availability

Not applicable.
